# Encephalopathy as a presenting feature of ascariasis in a child

**DOI:** 10.4103/0972-5229.78237

**Published:** 2011

**Authors:** Syed Ahmed Zaki, Vijay Lad

**Affiliations:** Department of Pediatrics, Lokmanya Tilak Municipal Medical College & General Hospital, Sion, Mumbai, India

Dear Editor,

A 5-year-old boy presented with two episodes of generalized tonic–clonic convulsions lasting for 5–10 minutes, without regaining consciousness. There was history of vague periumbilical abdominal pain since 1 day. There was no history of fever, vomiting, loose motions, head trauma, drug intake, past history of convulsions or contact with tuberculosis. On admission, he was unconscious with a Pediatric Glasgow coma scale score of 8/15 (E2 V2 M4). He was afebrile with normal vital parameters. His weight and length were below the 5 ^th^percentile, suggestive of malnutrition. All the deep tendon reflexes were depressed with bilateral extensor plantars. Rest of the central nervous system and other systemic examination was normal. Hemogram was normal except for low hemoglobin (8.6 g/dl). Liver and renal function tests, blood sugar, serum electrolytes, arterial blood gas analysis, urine examination and serum calcium were normal. Peripheral blood smear and rapid antigen test for malarial parasites were negative. Cerebrospinal fluid (CSF) examination including viral studies for herpes simplex and Japanese encephalitis were normal. Computed tomography scan and magnetic resonance imaging of the brain was normal. Stool examination revealed eggs of *Ascaris lumbricoides* HIV serology was negative. Ultrasonographic examination of abdomen revealed multiple worms in the jejunal lumen. He was started on intravenous fluids, loaded with intravenous phenytoin and was given piperazine citrate (150 mg/kg initially, followed by 65 mg/kg/dose BD for 3 days) through Ryle’s tube for 3 days. Blood culture and CSF culture showed no growth. The child passed several round worms in stool and vomitus on the 2nd day of admission. He improved and regained his normal sensorium within 72 hours of admission. He did not receive any other antibiotics or antiviral drugs. After excluding other causes of encephalopathy and response to anti-helminthic treatment, the diagnosis considered was acute encephalopathy secondary to ascariasis. He was discharged after 5 days of admission. Electroencephalogram done after 10 days was normal and phenytoin was tapered and omitted. On follow-up after 6 months, he is symptom-free and well.

The clinical presentation of ascariasis depends on the intensity of infection and the organs involved.[1] Although most individuals are asymptomatic, few can present acutely with intestinal obstruction and extra-intestinal complications.[1–2] Extra-intestinal complications can involve the pulmonary, hepato-biliary, renal and central nervous systems.[1–3] Encephalopathy as a presenting feature of ascariasis is unusual and rarely described.[3–5] Bapat *et al*. reported a 4-year-old male who developed encephalopathy and obstructive jaundice due to *Ascaris*.[3] He was treated with mebendazole with rapid improvement in sensorium. Similarly, Selimoglu *et al* and Jat *et al* have reported *Ascaris* encephalopathy in a 3.5-year-old girl and an 18-month-old boy, respectively.[4,5] Both the patients had history of passing *Ascaris* worms in their vomitus prior to admission. In addition, the boy also had abdominal distension and a lump palpable around the umbilicus. Ultrasonography of the abdomen revealed worms in the jejunal lumen. Both the patients were treated with pyrantel pamoate with rapid improvement in sensorium. The diagnosis in the above cases was established by excluding other causes of encephalopathy and by the response to antihelminthic drugs. The pathophysiology of encephalopathy in ascariasis still remains unclear. Various hypotheses have been put forward including the adverse effects of toxins produced by the larval or adult worms or their metabolites like acetaldehyde.[3–5] Another hypothesis postulated is an immune mechanism involving an antigen–antibody type of reaction in hypersensitive nervous tissue.[3–5] Encephalopathy due to ascariasis should be managed with supportive treatment and anti-helminthic drugs. The various anti-helminthic drugs used in the treatment of ascariasis include albendazole, mebendazole, pyrantel pamoate and piperazine citrate.[1,5]

Ascariasis is a common problem in children in tropical areas, particularly in the malnourished children. Physicians should be aware of this unusual presentation of ascariasis and should consider it in the differential diagnosis of unexplained encephalopathy.
